# Correlation Between Phase Angle and Body Composition, Strength and Nutritional Habits in Male Gamers

**DOI:** 10.3390/sports13080257

**Published:** 2025-08-06

**Authors:** Catarina N. Matias, Francesco Campa, Joana Cardoso, Margarida L. Cavaca, Rafael Carlos, Filipe J. Teixeira

**Affiliations:** 1 CIDEFES—Universidade Lusófona & CIFI2D—Universidade do Porto, Campo Grande nº 380, 1700-097 Lisboa, Portugal; p5262@ulusofona.pt; 2 Department of Biomedical Sciences, University of Padua, 35131, Padova, Italy; francesco.campa@unipd.it; 3 University of Maia, Av. Carlos de Oliveira Campos, 4475-690 Maia, Portugal; joanacardoso@umaia.pt; 4 Center for Psychology at the University of Porto, Rua Alfredo Allen, 4200-135 Porto, Portugal; 5 Faculdade de Motricidade Humana, Universidade de Lisboa, Estrada da Costa, 1495-751 Cruz-Quebrada, Portugal; margarida.slcavaca@gmail.com; 6 Atlântica—Instituto Universitário, Fábrica da Pólvora de Barcarena, 2730-036 Barcarena, Portugal; rafamcarlos@gmail.com; 7 Interdisciplinary Center for the Study of Human Performance (CIPER), Faculdade de Motricidade Humana, Universidade de Lisboa, Estrada da Costa, 1495-751 Cruz-Quebrada, Portugal; 8 Bettery Lifelab, Bettery S.A., Lagoas Park, Edificio 15, Piso 1, 2740-262 Porto Salvo, Portugal

**Keywords:** video games, nutrition, phase angle, body composition, strength

## Abstract

Gaming has evolved into a cultural phenomenon with a global reach, captivating millions of individuals. Nevertheless, little is known about this population. We aim to physiologically characterise the Portuguese gamers, bearing in mind that phase angle (PhA) is a general indicator of health, to check possible correlations between body composition, strength, and nutrition. A sample of 35 male gamers (individuals who play video games) was evaluated for anthropometry; body composition through DXA for whole-body bone mineral content (BMC), fat-free mass (FFM, kg), fat mass, and visceral adipose tissue, and through BIA (bioelectrical impedance analysis) for total body water (TBW), water pools (extracellular water and intracellular water, ICW), and PhA; strength through maximal isometric handgrip strength using a dynamometer; and nutritional intake using a three-day food record. Results show that participants are within reference metrics for all the analysed variables except regarding protein and carbohydrate intake (all values are above and below the Acceptable Macronutrient Distribution Ranges, respectively). A positive correlation was observed between PhA and TBW, ICW, handgrip strength, BMC and FFM, and a negative correlation with fat mass (absolute, percentage and visceral). In conclusion, PhA correlates with body composition variables, which aligns with previous research as a predictor of health and performance.

## 1. Introduction

In recent years, gaming has evolved into a global cultural phenomenon, captivating millions of individuals across various age groups [1]. Gamers, defined as individuals who play video games, are often characterised by exceptional reflexes, strategic thinking, and digital dexterity, making them a distinctive population and a compelling subject for scientific inquiry [2]. As gaming continues to establish itself as a mainstream form of entertainment and competition, the scientific community has shown increasing interest in exploring the complexities of this digital domain, ushering in a new era of interdisciplinary research. Both professional (i.e., gamers who make a career out of playing video games [3]) and amateur players invest significant time in honing their skills, often spending extended hours in front of screens. This has led to a growing awareness of the potential implications for physical health and well-being [4]. Consequently, current research is increasingly focused on the intricate relationships among gaming, body composition, and nutritional habits [5], recognising that optimal cognitive performance and sustained energy are essential for peak gameplay [6].

Dual-energy X-ray absorptiometry (DXA) is one of the most widely used reference methods for assessing body composition in laboratory settings. It provides precise measurements of bone mineral content and density, as well as fat-free mass (FFM) and fat mass (FM), offering a comprehensive multicomponent analysis of body mass [7]. However, despite its accuracy, DXA has limitations related to radiation exposure, lack of portability, and high cost. In response to these constraints, alternative methods such as bioimpedance have gained increasing attention [8]. Bioelectrical Impedance Analysis (BIA) is a non-invasive technique that measures the opposition to the flow of an electric current through body tissues, and it has emerged as a validated and practical alternative for assessing body composition [9]. Among BIA-derived parameters, phase angle (PhA), a direct measure based on raw impedance data, has recently garnered interest for its ability to reflect cellular integrity and membrane capacitance. PhA is positively associated with the intracellular-to-extracellular water ratio and muscle mass, and it shows strong correlations with physical performance metrics [10]. Furthermore, PhA is recognised as a robust indicator of cellular health, encompassing aspects such as membrane integrity, intracellular hydration, and oxidative or inflammatory status [11], which traditional anthropometric measures (e.g., BMI, FM, FFM) cannot capture. Importantly, PhA also offers advantages in terms of interpretability, numerical simplicity, and practical utility for longitudinal monitoring—while retaining meaningful biological and clinical relevance [12].

While extended hours spent playing electronic games may be associated with potentially adverse effects on body composition and overuse injuries, such as carpal tunnel syndrome, postural issues leading to back pain, and lower-body muscle atrophy [13], it is equally important to acknowledge the contrasting positive impact on hand strength [14]. Our group’s previous research [15] demonstrated that PhA was significantly associated with both muscle quantity and strength in overweight former athletes, even after adjusting for lean and fat mass. The intricate and demanding nature of certain video game genres, particularly those requiring rapid and precise hand movements, may inadvertently promote the development of hand strength and fine motor skills [6,14]. This dual-sided impact highlights the complexity of assessing gaming’s effects on body composition, muscular strength, and broader health-related parameters. It calls for a balanced evaluation that considers both the potential benefits and the risks associated with prolonged gaming.

By exploring the relationships between bioelectrical parameters, reflecting overall health, body composition, and the often-overlooked positive influence on handgrip strength, we aim to provide a comprehensive overview of the physiological profile of gamers. Our objective is to describe key physiological characteristics and identify meaningful associations within this rapidly expanding digital culture. More specifically, this study seeks to physiologically characterise the Portuguese gamer population, with particular emphasis on PhA as a general indicator and sensitive marker of health. Additionally, we aim to investigate potential associations among body composition, muscular strength, and nutritional factors.

## 2. Materials and Methods

### 2.1. Participants

From the 235 participants of the online survey (valid answers collected through Google Forms) conducted to characterise gaming habits and the profile of Portuguese gamers abroad [16], 35 participants volunteered to participate in this laboratory phase. Participants’ criteria for inclusion in this phase were (a) having a valid survey answer and (b) being available to visit our laboratory facilities (Lisbon, Portugal). This investigation received approval from the Universidade da Maia Ethics Committee Review Board (approval number 52/2021, date 5 June 2021) and adhered to all the standards of human research outlined in the Declaration of Helsinki [17]. Before enrolling on the study procedures, a full explanation of the study purposes, design, data collection procedures, and possible risks and benefits was provided. All participants provided both verbal and written informed consent before their enrolment.

### 2.2. Study Design

The online survey for the primary study was disseminated through the usual communication and social networks [16]. Participants who wanted to participate in the study’s laboratory analysis signed up at the end of this survey and were contacted via email to schedule a visit.

### 2.3. Assessments

Evaluations were performed at our laboratory early in the morning (08 h) after a 12 h fast and without consumption of alcohol or caffeine/stimulant beverages and at least 12 h from the last exercise session. Participants were asked discrete questions regarding their playing time, additional screen time, and physical activity habits. Measurements were performed as stated in the following sections.

#### 2.3.1. Anthropometry

Participants had their body weight and height measured wearing minimal clothing and without shoes to the nearest 0.1 kg and 0.1 cm, respectively, with a scale and a stadiometer (Seca, Hamburg, Germany) using standardised procedures as reported elsewhere [18].

#### 2.3.2. Body Composition

Body composition was determined using the following methods:

− Dual-energy X-ray absorptiometry (DXA) device (Horizon Wi, Hologic, Waltham, MA, USA) where participants underwent a whole-body scan according to the procedures recommended by the manufacturer [19]. The same technician positioned the patient, performed the scan, and executed the analyses. Measurements included whole-body bone mineral content (BMC, kg), FFM (kg), and FM (% and kg). Within FM, visceral adipose tissue was further analysed using DXA software Version 13.6.0.7:5 (VAT, cm^2^) [20].
− BIA was performed using a single frequency of 50 kHz device (BIVA PRO, Akern, Pisa, Italy) for estimates of whole-body resistance (R) and reactance (Xc). Assessments were obtained after a 10 min rest period in a supine position. From the raw data R and Xc, total body water (TBW) and water pools (extracellular water, ECW (L), and intracellular water, ICW (L)) were determined using Akern Software (version 1.19.2). Also, phase angle (PhA) was calculated as the arctangent of Xc/R × 180/π from R and Xc [21].

#### 2.3.3. Handgrip Strength

Both hands’ maximal isometric handgrip strength was determined alternately using a Jamar^®^ hydraulic hand dynamometer (Jamar, Sammons Preston, Inc., Bolingbrook, IL, USA). The participants were tested while standing with the elbow in full extension [22] and asked to squeeze the dynamometer at maximal effort for three trials, with a 30 s break between each trial. The maximal force was considered for analysis.

#### 2.3.4. Diet Control

Three-day food records (2 weekdays and 1 weekend day) were requested to characterise the participants’ food intake. After reporting the intake, a registered nutritionist analysed participants’ diary food logs and filled in gaps and lapses via individual interviews (phone calls) with the participants. Food records were then analysed by software (Nutritics Research Edition (v5.09), Dublin, Ireland) for total energy and macronutrient intake.

### 2.4. Statistical Analysis

The data was analysed using IBM SPSS Statistics version 28.0, 2012 (IBM, Chicago, IL, USA). The Kolmogorov–Smirnov test was applied in order to verify the normality of the variables. Basic frequency and descriptive statistics were run to characterise participants’ data. One sample *t*-test was performed to determine whether the mean calculated from the sample differed from the reference value. Bivariate and partial correlations were tested between all variables of interest. To better isolate the direct associations between phase angle, body composition, and physical performance outcomes, partial correlation analyses were conducted, controlling for fat-free mass (FFM), weekly time spent in physical activity, and self-reported playing time. These covariates were selected based on their known influence across all studied domains, as supported by previous research [23,24,25,26]. The significance for α was set at *p* ≤ 0.05.

## 3. Results

Participants responded to a full-length questionnaire, including a comprehensive set of sociodemographic data, games and physical activity patterns, nutritional and sleeping habits, and mental health and well-being, at a unique evaluation moment of their choice. The results of this questionnaire were fully analysed, with the information being described elsewhere [16]. For this investigation, the data from the questionnaire (gaming and physical activity patterns) concerns only 35 participants engaged in the laboratory phase, as shown in Table 1. All participants were men between 19 and 46 years old. Players (57.1% amateurs) reported FIFA and League of Legends as the most played games (34.3% and 28.6%, respectively), with a mean playing time of 4.1 ± 2.4 h.day^−1^, ranging from 1 to 10 h.day^−1^. Furthermore, they reported spending an additional 2.3 ± 0.8 h.day^−1^ of exposure time to the screen regarding other tasks excluding gaming (e.g., school or work-related). They also reported engaging in physical activity or exercise, with 74.3% (N = 26) reporting daily physical activity. From those 26 participants, the most reported exercise was soccer and futsal (40% of the sample), followed by resistance exercise training (17.1%), with a mean engagement time per week of 285.5 ± 198.8 min.week^−1^. Table 1 reports players’ physical characteristics and reference values for each variable.

Regarding the characteristics where a reference or cutoff value exists, one can verify that participants are within reference metrics for all the analysed variables except for protein and carbohydrate intake (all values are above and below the Acceptable Macronutrient Distribution Ranges (AMDR), respectively).

Correlations between phase angle and all the other variables were performed and are presented in Table 2.

A positive correlation was observed between phase angle and TBW, ICW, handgrip strength, BMC and FFM, and a negative correlation with fat mass (absolute, percentage and visceral). When controlling for FFM, the PhA-FM correlations were lost. Still, all the initial correlations remained significant when controlling for physical activity (min.week^−1^) and playing time (h.day^−1^).

## 4. Discussion

As the popularity of video gaming continues to soar, so does the scientific research into this burgeoning field. Researchers are increasingly drawn to unravelling the cognitive, psychological, and physiological aspects of gaming, seeking to understand the intricate interplay between technology, human performance, and the dynamics of virtual competition [37,38,39,40,41]. This intersection of gaming and scientific inquiry holds promise for uncovering insights into neuroplasticity [42], skill acquisition [43], and the broader implications of immersive digital experiences on the human mind and body. Exploring questions surrounding body composition and posture-related issues, with the potential consequences of prolonged screen time, led researchers to shed light on how gamers’ lifestyles may influence their overall health [13,44].

The present study explores the intricate interplay between health-related bioelectrical parameters, body composition, and handgrip strength in the dynamic realm of gaming. Recognising the multifaceted impact of gaming on physical and psychological aspects, our study aims to contribute valuable insights into the physiological dimensions influenced by gaming. Therefore, we seek to enhance the understanding of how gaming influences various facets of the human body, fostering a more informed perspective on the implications and potential benefits of this modern form of entertainment. So, this research had a two-fold aim: (1) to characterise the Portuguese gamer population regarding physiologic characteristics and (2) to check possible correlations between phase angle and body composition, strength, and nutrition.

A plethora of research has been made available regarding gamers in recent years due to the growing interest in the recognised advantages and disadvantages of gaming skills and abilities [45]. Moreover, a lot has been scrutinised, especially concerning the mental state and well-being-related parameters of gamers. In this regard, researchers have explored personality characteristics [46], internet gaming disorder [47,48,49,50], addiction [51], and nutritional habits and their interplay with optimal cognitive function as paramount to peak gaming performance [6]. Also, the constant engagement of fingers and hand muscles during gameplay as an unintentional exercise has been studied, showing it can improve hand strength and coordination [6,14]. Grip strength is a validated proxy of overall neuromuscular function, correlated with hand-eye coordination, fine motor control, and reaction speed, critical components of gaming performance [52]. Importantly, grip strength serves as a functional measure of muscular health, supporting posture and ergonomic resilience during prolonged gaming sessions, and relates closely to PhA [15], reinforcing its role in characterising both bodily and cellular function.

Nevertheless, to our knowledge, there is no accurate and precise data on this population’s body composition, strength, or nutritional habits. Recently, a systematic review on the health consequences of intensive gaming [45] reinforced that although clinical guidelines have been established for a safe approach to gaming aiming to prevent and treat major physical and psychological illnesses, little is known regarding the actual characteristics of these players [53,54]. In line with the information above and to meet the study’s first aim, the present investigation brings some clarity around this topic since a small sample of Portuguese gamers were assessed in a laboratory setting regarding body composition, strength, and food habits/intake in their real-lifetime context, which adds ecological validity to our findings. Although the Portuguese gamers population is estimated to be approximately 1.9 million (non-official data), there are ~1000 professional and recreational gamers registered in the esports federation. A small sample of those volunteered to undergo laboratory evaluation to assess their characteristics further. Although our study did not include a non-gaming control group, existing comparative research offers useful context. Ketelhut et al. (2023) found no significant differences in VO_2_max, grip strength, physical activity levels, or BMI between competitive e-gamers and matched non-gamers [55]. In contrast, DiFrancisco-Donoghue et al. (2022) showed that collegiate esports players were significantly less active and had higher body fat percentage, lower lean body mass, and reduced bone mineral content compared to age- and sex-matched controls, despite similar BMI values [56]. Also, and more recently, DiFrancisco-Donoghue et al. [57] reinforced that male and female gamers exhibited lower regional lean mass (especially forearm and upper body), reduced grip strength, and higher musculoskeletal pain compared to controls matched for age and BMI, highlighting the heterogeneity within this population and questioning generalised health assumptions about gaming populations.

### 4.1. General Characteristics and Food Intake

Participants were within the reference values when anthropometry (body mass index; BMI), body composition (TBW, PhA, FM and VAT), and strength (handgrip on both sides) variables were evaluated. When food consumption was further analysed, participants displayed an intake below the minimum recommended values regarding carbohydrate intake and above the AMDR when considering protein intake. Although a registered nutritionist carefully assessed the food intake (food record), and the gaps and lapses were fully corrected in individual interviews (phone calls) with the participants, it has been shown that food records tend to underreport macronutrient and energy intake, with protein being the least underreported macronutrient [58]. Albeit higher than the AMDR, our findings regarding reported protein intake align with the last national food intake inquiry to the Portuguese population (19.9% of total daily energy intake from protein) [59]. These results contrast with a study [60] where e-gamers reported a mean daily protein intake of less than 39.3 g than reported in our work and an equal carbohydrate intake (210 g). In light of the current evidence, we cannot assert macronutrient adequacy. Other authors have claimed that there is an urgent need to establish recommendations for this population [61], and this is further supported by the fact that only recently has the energy expenditure of the said athletes been analysed [62]. Nonetheless, it has been theorised that higher protein diets, like the one reported in the Portuguese population and in our work, could be beneficial for esports [61].

### 4.2. Body Composition and Phase Angle

Correlations between phase angle, body composition variables, and strength were further scrutinised to answer the second proposed goal. Our findings demonstrate that a higher PhA is positively correlated with FFM and handgrip strength and negatively correlated with total body fat. These relationships hold even after controlling for physical activity habits and gaming time, underscoring the direct relevance of PhA as an indicator of underlying physiological status. As is known, PhA reflects cell membrane integrity, intracellular water content, and muscle quality, and higher PhA values have been linked to greater muscle strength and lower incidence of muscle dysfunction. In our own group’s work, Matias et al. (2021) demonstrated that PhA is significantly associated with both muscle quantity and strength in overweight former athletes, even when adjusted for lean mass and fat mass [15]. Consistent with these findings, our results in e-gamers, where higher PhA correlated with greater FFM and stronger handgrip, suggest that PhA may serve as a valuable marker of muscle quality status in this population, despite their lifestyle. From a health standpoint, lower PhA values are associated with elevated body fat and poorer nutritional status and are often linked to reduced membrane integrity, inflammation, and increased risk for metabolic and cardiovascular diseases [63]. Our previous findings in the same population [16] demonstrated that prolonged gaming time was associated with poorer nutritional habits, disrupted sleep hygiene, and lower mental health scores, all of which have implications for overall health status. From a real-world perspective within gaming populations, where prolonged sitting time is pervasive, monitoring PhA could help identify individuals at higher risk for adverse metabolic changes and facilitate early lifestyle interventions. These findings reinforce the role of PhA as a valuable marker in identifying individuals at risk due to digital and sedentary lifestyles.

From the viewpoint of performance, while gaming is not physically demanding in the traditional sense, as it is primarily cognitive, grip strength and muscle condition, ergonomic endurance, neuromuscular coordination, and postural resilience are critical to support prolonged posture, reaction speed, and injury prevention during gaming sessions. Interventions that increase PhA, may therefore contribute to improved ergonomic resilience, improved physical readiness and overall well-being among gamers.

Additionally, one should consider that these correlations remained significant even when controlling for physical activity habits (285.5 ± 198.8 min.week^−1^ of physical activity practice, mainly soccer and futsal, and resistance exercise training). Physical activity has already been proposed as a possible solution regarding an ominous cycle between nutritional habits, mental health, and sleep hygiene observed in this population (according to Matias et al., currently under review). A negative correlation between sports practice and mental health, nutritional habits, and sleeping problems suggests that physical activity may act as a protective factor for the development and treatment of mental disorders. At the same time, physical activity seems also to be correlated with healthier nutritional habits and better sleep hygiene.

In line with the current evidence, PhA seemed to predict overall body composition and performance in gamers. The results of the present study show that phase angle is positively correlated with handgrip strength and FFM, both fundamental markers to attain optimal levels of physical performance. Additionally, low phase angle values were correlated with higher total body fat levels, regardless of playing time and weekly sports activity levels. The average PhA measured in this study appears to be lower when compared to the 50th percentile, as suggested by a recent study that provided reference values for the general healthy population [10]. Specifically, while 7.3° is the mean value at the 50th percentile for male subjects, participants in this study align with values of the 25th percentile. We posit that this lower value may be attributed to a prolonged sedentary condition resulting from medium- to long-term engagement in gaming activities. These findings suggest that monitoring phase angle may provide essential insights into the overall health of this population, which may be at risk due to prolonged periods of sedentary behaviour. Furthermore, it is a simple and relatively affordable measurement that allows for periodic monitoring and serves as a non-invasive and swift assessment when using bioelectrical impedance analysis. Through this study, we seek to expand the field of knowledge about the gamers population and provide valuable insights into the broader implications of digital lifestyles on physical health-related parameters.

### 4.3. Limitations Section

Although this manuscript presents several novelties regarding the gamer population and their unique characteristics, one must acknowledge some limitations of this investigation and suggest solutions for future research. Regarding sampling, the sample size is small due to logistics, scheduling, and the distance to the laboratory. Although this study offers novel laboratory-based insights into the physiological characteristics of gamers, the small sample size in this phase limits the statistical power and overall generalisability of the findings. As such, results should be interpreted as exploratory and hypothesis-generating rather than conclusive. Future studies with larger and more diverse cohorts are required to validate and extend these observations. Also, it needs to be addressed that only males were considered; therefore, future research should also include female gamers. It should also be considered that although this study did not include a matched control group, comparisons with age- and sex-specific normative data were used to contextualise the findings. This approach, while limited, allowed us to identify potential deviations from expected values and highlight physiological patterns specific to gamers. Considering the methodological approach, we acknowledge that DXA scans could be explicitly performed on bone-sensitive areas such as the collum and/or femur to address postural issues and reinforce bone quality; also, water pool variations may have generated bias due to the inherent limitations of bioimpedance analysis; thus, future analysis should use more sensitive methods to control for water pools (i.e., labelled water techniques). An additional limitation of the present study is that no correction for multiple comparisons was applied to the correlation analyses. However, given the exploratory and hypothesis-driven nature of this investigation (explained in the statistics section), our primary aim was to detect potentially meaningful associations between physiological markers within a relatively small and specific population. In future studies with larger sample sizes and expanded variable sets, correction methods for multiple testing (e.g., Bonferroni) should be implemented to improve statistical robustness and confirm the present findings.

### 4.4. Clinical Recommendations

Regular physical activity should be encouraged in gamers, with at least 150 min of moderate-intensity exercise per week, including both cardiovascular and resistance training. Additionally, handgrip strength exercises can enhance dexterity and reduce the risk of musculoskeletal issues related to prolonged gaming sessions.

Gamers should monitor their phase angle (PhA) regularly, as it serves as a useful predictor of overall health and performance. A lower PhA may indicate prolonged sedentary behaviour and suboptimal body composition, reinforcing the importance of tracking this metric over time.

A balanced macronutrient intake and adequate hydration are crucial to maintaining energy levels and overall health [36]. A higher protein intake has been theorised to be advantageous for e-gamers, although the current evidence does not allow us to establish a specific protein recommendation [61].

## 5. Conclusions

The present study addresses the body composition, strength, and nutritional habits of a Portuguese gamer population. This population does not differ from general populations regarding body composition or strength values. However, insofar as self-reported protein and carbohydrate intake, our results are above and below the Acceptable Macronutrient Distribution Ranges (AMDR), respectively. Phase angle correlated with body composition variables, again showing its usefulness as a predictor of health and performance.

## Figures and Tables

**Table 1:** Characteristics of the players.

	Minimum	Maximum	Mean (SD)	Reference Value
Age (years)	19.4	45.9	25.3 ± 6.0	--
Height (cm)	166.5	187.0	175.9 ± 5.2	--
Weight (kg)	58.6	113.3	75.6 ± 12.8	--
BMI (kg.m^−2^) *^1^	19.5	36.7	24.4 ± 4.0	18.5–24.9
TBW (L) *^2^	34.3	52.1	43.3 ± 5.3	39–52.4
ECW (L)	15.1	24.3	18.4 ± 2.1	--
ICW (L)	18.2	31.6	24.9 ± 3.7	--
PhA (º) *^3^	5.4	8.3	6.8 ± 0.7	>6
Handgrip right side (kg.F) *^4^	25.0	71.0	46.5 ± 10.2	>45
Handgrip left side (kg.F) *^4^	26.0	70.0	43.8 ± 9.5	>42
BMC (kg)	1.7	3.5	2.7 ± 0.4	--
FFM (kg)	42.6	68.6	56.4 ± 7.0	--
FM (kg)	7.9	48.9	17.5 ± 9.7	--
FM (%) *^5^	11.9	44.7	22.8 ± 8.6	10.5–21.8
Visceral Fat area (cm^2^) *^6^	39.3	180.7	74.2 ± 33.5	10–100
Energy (kcal)	1551.4	3437.3	2108.5 ± 410.2	
Energy (kcal/kg body weight)	13.7	55.4	28.8 ± 8.2	
Protein consumption (g)	72.1	185.3	119.2 ± 29.8	
Protein consumption (%) *^7^	12.7	36.5	22.9 ± 5.3	10–15 ^#^
Fat consumption (g)	54.8	134.1	83.4 ± 20.2	
Fat consumption (%) *^7^	20.8	38.3	27.8 ± 4.8	15–30
Carbohydrate consumption (g)	95.9	462.3	210.2 ± 63.4	
Carbohydrate consumption (% *) *^7^	23.6	53.8	39.5 ± 6.7	55–75 ^#^

*^1^ [27,28]; *^2^ [29]; *^3^ [30]; *^4^ [22]; *^5^ [31,32]; *^6^ [33,34,35]; *^7^ [36]. ^#^ significantly different from reference. Abbreviations: BMI, body mass index; TBW, total body water; ECW, extracellular water; ICW, intracellular water; PhA, phase angle; BMC, bone mineral content; FFM, fat-free mass; FM, fat mass.

**Table 2:** Bivariate Correlation (panel a) and partial correlations (panel b) (N = 35).

		Panel A		Panel B					
		Bivariate Correlation		Controlling for FFM		Controlling for Physical Activity Practice (min.week^−1^)		Controlling for Playing Time (h.day^−1^)	
		r	*p*	r	*p*	r	*p*	r	*p*
PhA	TBW	0.437	0.009	--	--	0.380	0.050	0.405	0.018
	ICW	0.686	<0.001	0.622	<0.001	0.680	0.000	0.651	0.000
	Handgrip right side	0.629	<0.001	0.433	0.011	0.652	0.000	0.599	0.000
	Handgrip left side	0.607	<0.001	0.419	0.014	0.628	0.000	0.606	0.000
	BMC	0.710	<0.001	--	--	0.348	0.075	0.437	0.010
	FFM	0.523	0.001	--	--	0.478	0.012	0.484	0.004
	FM (absolute)	−0.348	0.040	−0.480	0.004	−0.331	0.092	−0.317	0.067
	FM (%)	−0.522	0.001	−0.519	0.002	−0.468	0.014	−0.489	0.003
	VAT (cm^2^)	−0.252	0.040	−0.362	0.035	−0.228	0.252	−0.247	0.158

Abbreviations: PhA, phase angle; TBW, total body water; ICW, intracellular water; BMC, bone mineral content; FFM, fat-free mass; VAT, visceral adipose tissue; FM, fat mass.

## Data Availability

The datasets used and analysed during the current study are available from the corresponding author upon reasonable request.

## Abbreviations

The following abbreviations are used in this manuscript:

AMDR	Acceptable Macronutrient Distribution Ranges
BIA	Bioelectrical Impedance Analysis
BIVA	Bioelectrical Impedance Vector Analysis
BMC	Bone Mineral Content
BMI	Body Mass Index
DXA	Dual-energy X-ray Absorptiometry
ECW	Extracellular Water
FFM	Fat-Free Mass
FM	Fat Mass
ICW	Intracellular Water
PhA	Phase Angle
TBW	Total Body Water
VAT	Visceral Adipose Tissue

## References

[1] Limone P., Ragni B., Toto G.A. (2023). The epidemiology and effects of video game addiction: A systematic review and meta-analysis. Acta Psychol..

[2] Yin K., Zi Y., Zhuang W., Gao Y., Tong Y., Song L., Liu Y. (2020). Linking Esports to health risks and benefits: Current knowledge and future research needs. J. Sport Health Sci..

[3] Adams T. (2012). Playing computer games as electronic sport: In search of a theoretical framework for a new research field. Computer Games and New Media Cultures: A Handbook of Digital Games Studies.

[4] Kelly S., Leung J. (2021). The New Frontier of Esports and Gaming: A Scoping Meta-Review of Health Impacts and Research Agenda. Front. Sports Act. Living.

[5] Chan G., Huo Y., Kelly S., Leung J., Tisdale C., Gullo M. (2022). The impact of eSports and online video gaming on lifestyle behaviours in youth: A systematic review. Comput. Human. Behav..

[6] Thomas C.J., Rothschild J., Earnest C.P., Blaisdell A. (2019). The Effects of Energy Drink Consumption on Cognitive and Physical Performance in Elite League of Legends Players. Sports.

[7] Dallman J., Herda A., Cleary C.J., Morey T., Diederich A., Vopat B.G., Vopat L.M. (2024). A Brief Review of the Literature for Published Dual-Energy X-Ray Absorptiometry Protocols for Athletes. Sports Health.

[8] Campa F., Gatterer H., Lukaski H., Toselli S. (2019). Stabilizing Bioimpedance-Vector-Analysis Measures With a 10-Minute Cold Shower After Running Exercise to Enable Assessment of Body Hydration. Int. J. Sports Physiol. Perform..

[9] Campa F., Toselli S., Mazzilli M., Gobbo L.A., Coratella G. (2021). Assessment of Body Composition in Athletes: A Narrative Review of Available Methods with Special Reference to Quantitative and Qualitative Bioimpedance Analysis. Nutrients.

[10] Campa F., Coratella G., Cerullo G., Stagi S., Paoli S., Marini S., Grigoletto A., Moroni A., Petri C., Andreoli A. (2023). New bioelectrical impedance vector references and phase angle centile curves in 4,367 adults: The need for an urgent update after 30 years. Clin. Nutr..

[11] da Silva B.R., Orsso C.E., Gonzalez M.C., Sicchieri J.M.F., Mialich M.S., Jordao A.A., Prado C.M. (2023). Phase angle and cellular health: Inflammation and oxidative damage. Rev. Endocr. Metab. Disord..

[12] Ward L.C., Brantlov S. (2023). Bioimpedance basics and phase angle fundamentals. Rev. Endocr. Metab. Disord..

[13] DiFrancisco-Donoghue J., Balentine J., Schmidt G., Zwibel H. (2019). Managing the health of the eSport athlete: An integrated health management model. BMJ Open Sport Exerc. Med..

[14] Bostanci H., Emir A., Tarakci D., Tarakci E. (2020). Video game-based therapy for the non-dominant hand improves manual skills and grip strength. Hand Surg. Rehabil..

[15] Matias C.N., Campa F., Nunes C.L., Francisco R., Jesus F., Cardoso M., Valamatos M.J., Homens P.M., Sardinha L.B., Martins P. (2021). Phase Angle Is a Marker of Muscle Quantity and Strength in Overweight/Obese Former Athletes. Int. J. Environ. Res. Public Health.

[16] Matias C.N., Cardoso J., Cavaca M.L., Cardoso S., Giro R., Vaz J., Couto P.A., Dores A.R., Ferreira T.B., Tinsley G.M. (2023). Game on: A cross-sectional study on gamers’ mental health, Game patterns, physical activity, eating and sleeping habits. Comput. Human. Behav..

[17] World Medical A. (2013). World medical association declaration of helsinki: Ethical principles for medical research involving human subjects. JAMA.

[18] Lohman T.G., Roche A.F., Martorell R. (1988). Anthropometric Standardization Reference Manual.

[19] Hologic (2020). Horizon Series-Operator Manual.

[20] Gadekar T., Dudeja P., Basu I., Vashisht S., Mukherji S. (2020). Correlation of visceral body fat with waist-hip ratio, waist circumference and body mass index in healthy adults: A cross sectional study. Med. J. Armed Forces India.

[21] Matias C.N., Campa F., Cerullo G., D’Antona G., Giro R., Faleiro J., Reis J.F., Monteiro C.P., Valamatos M.J., Teixeira F.J. (2022). Bioelectrical Impedance Vector Analysis Discriminates Aerobic Power in Futsal Players: The Role of Body Composition. Biology.

[22] Spruit M.A., Sillen M.J., Groenen M.T., Wouters E.F., Franssen F.M. (2013). New normative values for handgrip strength: Results from the UK Biobank. J. Am. Med. Dir. Assoc..

[23] Langer R.D., Guimarães R.F., Guerra-Júnior G., Gonçalves E.M. (2022). Can Phase Angle Be Associated With Muscle Strength in Healthy Male Army Cadets?. Mil. Med..

[24] Mundstock E., Amaral M.A., Baptista R.R., Sarria E.E., Dos Santos R.R.G., Filho A.D., Rodrigues C.A.S., Forte G.C., Castro L., Padoin A.V. (2019). Association between phase angle from bioelectrical impedance analysis and level of physical activity: Systematic review and meta-analysis. Clin. Nutr..

[25] Yamada Y., Yoshida T., Murakami H., Kawakami R., Gando Y., Ohno H., Tanisawa K., Konishi K., Julien T., Kondo E. (2022). Phase angle obtained via bioelectrical impedance analysis and objectively measured physical activity or exercise habits. Sci. Rep..

[26] Lin L.Y., Chen J., Lai T.F., Chung Y.Y., Park J.H., Hu Y.J., Liao Y. (2023). Sedentary Behavior and Phase Angle: An Objective Assessment in Physically Active and Inactive Older Adults. Nutrients.

[27] Quetelet A. (1832). Recherches sur le poids de l’ homme aux different âges. Nouveaux Memoire de l’ Academie Royale des Sciences et BellesLettres de Bruxelles.

[28] Berg J., Nauman J., Wisløff U. (2024). Normative values for body composition in 22,191 healthy Norwegian adults 20–99 years: The HUNT4 study. Prog. Cardiovasc. Dis..

[29] Chumlea W.C., Guo S.S., Zeller C.M., Reo N.V., Baumgartner R.N., Garry P.J., Wang J., Pierson R.N., Heymsfield S.B., Siervogel R.M. (2001). Total body water reference values and prediction equations for adults. Kidney Int..

[30] Mattiello R., Amaral M.A., Mundstock E., Ziegelmann P.K. (2020). Reference values for the phase angle of the electrical bioimpedance: Systematic review and meta-analysis involving more than 250,000 subjects. Clin. Nutr..

[31] Pasco J.A., Holloway K.L., Dobbins A.G., Kotowicz M.A., Williams L.J., Brennan S.L. (2014). Body mass index and measures of body fat for defining obesity and underweight: A cross-sectional, population-based study. BMC Obes..

[32] Peterson M.J., Czerwinski S.A., Siervogel R.M. (2003). Development and validation of skinfold-thickness prediction equations with a 4-compartment model. Am. J. Clin. Nutr..

[33] Brochu M., Tchernof A., Turner A.N., Ades P.A., Poehlman E.T. (2003). Is there a threshold of visceral fat loss that improves the metabolic profile in obese postmenopausal women?. Metabolism.

[34] Pickhardt P.J., Jee Y., O’Connor S.D., del Rio A.M. (2012). Visceral adiposity and hepatic steatosis at abdominal CT: Association with the metabolic syndrome. AJR. Am. J. Roentgenol..

[35] Nicklas B.J., Penninx B.W., Ryan A.S., Berman D.M., Lynch N.A., Dennis K.E. (2003). Visceral adipose tissue cutoffs associated with metabolic risk factors for coronary heart disease in women. Diabetes Care.

[36] WHO (2003). Diet, Nutrition and the Prevention of Chronic Diseases.

[37] Franceschini S., Bertoni S., Lulli M., Pievani T., Facoetti A. (2022). Short-Term Effects of Video-Games on Cognitive Enhancement: The Role of Positive Emotions. J. Cogn. Enhanc..

[38] Comeras-Chueca C., Marin-Puyalto J., Matute-Llorente A., Vicente-Rodriguez G., Casajus J.A., Gonzalez-Aguero A. (2021). Effects of Active Video Games on Health-Related Physical Fitness and Motor Competence in Children and Adolescents With Overweight or Obesity: Systematic Review and Meta-Analysis. JMIR Serious Games.

[39] West R., Swing E.L., Anderson C.A., Prot S. (2020). The Contrasting Effects of an Action Video Game on Visuo-Spatial Processing and Proactive Cognitive Control. Int. J. Environ. Res. Public Health.

[40] Alsaad F., Binkhamis L., Alsalman A., Alabdulqader N., Alamer M., Abualait T., Khalil M.S., Al Ghamdi K.S. (2022). Impact of Action Video Gaming Behavior on Attention, Anxiety, and Sleep Among University Students. Psychol. Res. Behav. Manag..

[41] Leis O., Lautenbach F. (2020). Psychological and physiological stress in non-competitive and competitive esports settings: A systematic review. Psychol. Sport Exerc..

[42] Iosa M., Verrelli C.M., Gentile A.E., Ruggieri M., Polizzi A. (2022). Gaming Technology for Pediatric Neurorehabilitation: A Systematic Review. Front. Pediatr..

[43] Zheng L., Oberle C., Hawkes-Robinson W., Daniau S. (2021). Serious Games as a Complementary Tool for Social Skill Development in Young People: A Systematic Review of the Literature. Simul. Gaming.

[44] Rudolf K., Bickmann P., Froböse I., Tholl C., Wechsler K., Grieben C. (2020). Demographics and Health Behavior of Video Game and eSports Players in Germany: The eSports Study 2019. Int. J. Environ. Res. Public Health.

[45] Shen Y., Cicchella A. (2023). Health Consequences of Intensive E-Gaming: A Systematic Review. Int. J. Environ. Res. Public Health.

[46] Sharma M.K., Anand N., Murthy K.D. (2020). Personality characteristics of online gamers. Indian J. Psychiatry.

[47] Gentile D.A., Bailey K., Bavelier D., Brockmyer J.F., Cash H., Coyne S.M., Doan A., Grant D.S., Green C.S., Griffiths M. (2017). Internet Gaming Disorder in Children and Adolescents. Pediatrics.

[48] Chung S.J., Jang J.H., Lee J.Y., Choi A., Kim B.M., Park M.K., Jung M.H., Choi J.S. (2020). Self-Efficacy and Clinical Characteristics in Casual Gamers Compared to Excessive Gaming Users and Non-Gamers in Young Adults. J. Clin. Med..

[49] Liao Z., Huang Q., Huang S., Tan L., Shao T., Fang T., Chen X., Lin S., Qi J., Cai Y. (2020). Prevalence of Internet Gaming Disorder and Its Association With Personality Traits and Gaming Characteristics Among Chinese Adolescent Gamers. Front. Psychiatry.

[50] Kim D., Nam J.K., Keum C. (2022). Adolescent Internet gaming addiction and personality characteristics by game genre. PLoS ONE.

[51] Geisel O., Panneck P., Stickel A., Schneider M., Müller C.A. (2015). Characteristics of Social Network Gamers: Results of an Online Survey. Front. Psychiatry.

[52] Ergezen G., Köşker A.B., Sözeri M.E., Okan M., İnan E. (2025). Effect of E-Sports Training on Hand Functions and Reaction Time in Young Adults: Randomized Controlled Study. J. Adv. Sport Technol..

[53] Franks R.R., King D., Bodine W., Chisari E., Heller A., Jamal F.t., Luksch J., Quinn K., Singh R., Solomon M. (2022). AOASM Position Statement on Esports, Active Video Gaming, and the Role of the Sports Medicine Physician. Clin. J. Sport Med..

[54] Emara A.K., Ng M.K., Cruickshank J.A., Kampert M.W., Piuzzi N.S., Schaffer J.L., King D. (2020). Gamer’s Health Guide: Optimizing Performance, Recognizing Hazards, and Promoting Wellness in Esports. Curr. Sports Med. Rep..

[55] Ketelhut S., Bodman A., Ries T., Nigg C.R. (2023). Challenging the Portrait of the Unhealthy Gamer-The Fitness and Health Status of Esports Players and Their Peers: Comparative Cross-Sectional Study. J. Med. Internet Res..

[56] DiFrancisco-Donoghue J., Werner W.G., Douris P.C., Zwibel H. (2022). Esports players, got muscle? Competitive video game players’ physical activity, body fat, bone mineral content, and muscle mass in comparison to matched controls. J. Sport Health Sci..

[57] DiFrancisco-Donoghue J., Jung M.-K., Balentine M.J., Zwibel H. (2025). Where Muscle Matters: How Regional Differences, Pain, and Gender Define Gamer Health. Int. J. Environ. Res. Public Health.

[58] Ravelli M.N., Schoeller D.A. (2020). Traditional Self-Reported Dietary Instruments Are Prone to Inaccuracies and New Approaches Are Needed. Front. Nutr..

[59] https://ian-af.up.pt/.

[60] Goulart J.B., Aitken L.S., Siddiqui S., Cuevas M., Cardenas J., Beathard K.M., Riechman S.E. (2023). Nutrition, lifestyle, and cognitive performance in esport athletes. Front. Nutr..

[61] Ribeiro F.J., Viana V.M., Borges N.P., Teixeira V.H. (2021). The Emergence of eSports Nutrition: A Review. Cent. Eur. J. Sport Sci. Med..

[62] Nicholson M., Poulus D., Robergs R., Kelly V., McNulty C. (2024). How Much Energy Do E’Athletes Use during Gameplay? Quantifying Energy Expenditure and Heart Rate Variability Within E’Athletes. Sports Med.—Open.

[63] Barbosa-Silva M.C., Barros A.J., Wang J., Heymsfield S.B., Pierson R.N. (2005). Bioelectrical impedance analysis: Population reference values for phase angle by age and sex. Am. J. Clin. Nutr..

